# Fabrication of Drug-Eluting Nano-Hydroxylapatite Filled Polycaprolactone Nanocomposites Using Solution-Extrusion 3D Printing Technique

**DOI:** 10.3390/polym13030318

**Published:** 2021-01-20

**Authors:** Pang-Yun Chou, Ying-Chao Chou, Yu-Hsuan Lai, Yu-Ting Lin, Chia-Jung Lu, Shih-Jung Liu

**Affiliations:** 1 Department of Mechanical Engineering, Chang Gung University, Taoyuan 33302, Taiwan; m7406@cgmh.org.tw (P.-Y.C.); s9409612563@gmail.com (Y.-H.L.); yutinna9876@mail.cgu.edu.tw (Y.-T.L.); happy2231017@mail.cgu.edu.tw (C.-J.L.); 2 Department of Plastic and Reconstructive Surgery and Craniofacial Research Center, Chang Gung Memorial Hospital, Taoyuan 33305, Taiwan; 3 Department of Orthopedic Surgery, Bone and Joint Research Center, Chang Gung Memorial Hospital-Linkou, Taoyuan 33305, Taiwan; enjoycu@ms22.hinet.net

**Keywords:** polycaprolactone, nano-hydroxylapatite, 3D printing, solution extrusion, process optimization, drug release

## Abstract

Polycaprolactone/nano-hydroxylapatite (PCL/nHA) nanocomposites have found use in tissue engineering and drug delivery owing to their good biocompatibility with these types of applications in addition to their mechanical characteristics. Three-dimensional (3D) printing of PCL/nHA nanocomposites persists as a defiance mostly because of the lack of commercial filaments for the conventional fused deposition modeling (FDM) method. In addition, as the composites are prepared using FDM for the purpose of delivering pharmaceuticals, thermal energy can destroy the embedded drugs and biomolecules. In this report, we investigated 3D printing of PCL/nHA using a lab-developed solution-extrusion printer, which consists of an extrusion feeder, a syringe with a dispensing nozzle, a collection table, and a command port. The effects of distinct printing variables on the mechanical properties of nanocomposites were investigated. Drug-eluting nanocomposite screws were also prepared using solution-extrusion 3D printing. The empirical outcomes suggest that the tensile properties of the 3D-printed PCL/nHA nanocomposites increased with the PCL/nHA-to-dichloromethane (DCM) ratio, fill density, and print orientation but decreased with an increase in the moving speed of the dispensing tip. Furthermore, printed drug-eluting PCL/nHA screws eluted high levels of antimicrobial vancomycin and ceftazidime over a 14-day period. Solution-extrusion 3D printing demonstrated excellent capabilities for fabricating drug-loaded implants for various medical applications.

## 1. Introduction

Degenerative pathologies, injuries, and trauma can harm bone tissues and lead to the requirement for therapies that facilitate their repair, replacement, or regeneration of the tissue. Scaffolds made of distinct biomaterials have been used as a substitute for bone regeneration. Bioceramic such as nano-hydroxyapatite (nHA) is known to promote cell proliferation and osteoconduction, and has been widely used as a bone graft substitute due to its good biocompatible and osteoconductive properties [1,2,3]. However, nHA possesses low mechanical properties because of its brittleness. Polycaprolactone (PCL), on the other hand, is a degradable polymer widely researched for use in long-term implants and controlled drug release applications [4]. nHA-filled PCL nanocomposites can be a good candidate as a synthetic alternative for bone tissue engineering and drug delivery, mainly owing to their excellent biocompatible and mechanical properties [5].

The 3D printing technique [6,7] is a novel technique for making unusual or complex component geometries that might be difficult to fabricate via other processes. The 3D printing process enables and facilitates the manufacture of moderate to mass numbers of parts that can be specifically customized. The technologies provide new opportunities with regard to the manufacturing paradigm and fabrication possibilities with substantially reduced times. New designs require only a short time to market, and customer demands can be fulfilled more rapidly.

Among various 3D printing methods, FDM is the most extensively used technique [8]. The procedure extrudes hot polymer melts from a nozzle and paves them on a collection table for product development. The extruding nozzle shifts in a horizontal position to form a single layer at a time after which the extruding nozzle moves consecutively in a vertical position so as to make a fresh layer. A computer is generally used to command the migration of the extrusion nozzle until a 3D-printed part is acquired.

The 3D printing of virgin PCL and composites [9,10] continues to be a defiance, largely because the filaments for FDM are generally limited [11,12]. Guerra and Ciurana [13] proposed a fused filament fabrication (FFF) printing device to print 3D stents out of PCL. Jhao et al. [14] explored hydroxyapatite/PCL scaffold printing using a lab-exploited melt-differential FDM printing facility. Hollander et al. [15] used FDM-printed PCL grafts to transport micronized indomethacin. Visscher et al. [16] integrated FDM and a salt leaching process for manufacturing a PCL scaffold of micro-/macro-porosity. In spite of these efforts, all developments rely on the FDM scheme that extrudes hot melted polymer during the printing process. The use of polymer melt extrusion, however, leads to some restrictions, specifically as the manufactured product is used for the intent of pharmaceutical delivery. Compounding or mixing drugs and hot polymer melt in the 3D extrusion printing process can damage or degenerate the pharmaceuticals [17].

One solution for coping with this concern is the use of the solution-extrusion 3D printing technique [18], which integrates a fluid-delivery unit and an automatized three-axis migration device for extrusion printing. To print a part, PCL, fillers, and solvent are primarily compounded and extruded from a feeding system consisting of a syringe equipped with a delivery nozzle. The nozzle is controlled and shifted by a computer/microprocessor. Once the solvent becomes volatile, the solution that is expelled from the delivery nozzle solidifies and forms successive layers to create 3D products of needed shapes.

This study exploited drug-eluting nHA-filled PCL nanocomposites using the solution-extrusion 3D printing technique so as to avoid the deactivation of embedded pharmaceuticals. An empirical study was completed to assess the effect of distinct printing variables on the tensile properties of 3D-printed PCL/nHA nanomaterials. The tensile strength of post-printed PCL/nHA specimens was estimated using a tensile test machine, and the morphological structure was examined via a field emission scanning electron microscope (FESEM) and a projector microscope. In addition, vancomycin- and ceftazidime-loaded PCL/nHA screws were also prepared using solution-extrusion 3D printing. Printed screws were assessed by differential scanning calorimeter (DSC) and Fourier-transform infrared (FTIR) spectroscopy. The elution characteristics of incorporated drugs were also evaluated via a high-performance liquid chromatography (HPLC).

## 2. Materials and Methods

### 2.1. Materials

PCL (*M*_n_: 80,000 Da), nHA (<200 nm, *M*_w_: 502.31 g/mol), and DCM were used for 3D printing, and vancomycin hydrochloride and ceftazidime hydrate were used as pharmaceuticals. All of them were acquired from Sigma-Aldrich (St. Louis, MO, USA).

### 2.2. Experimental Methods

**Figure 1:**
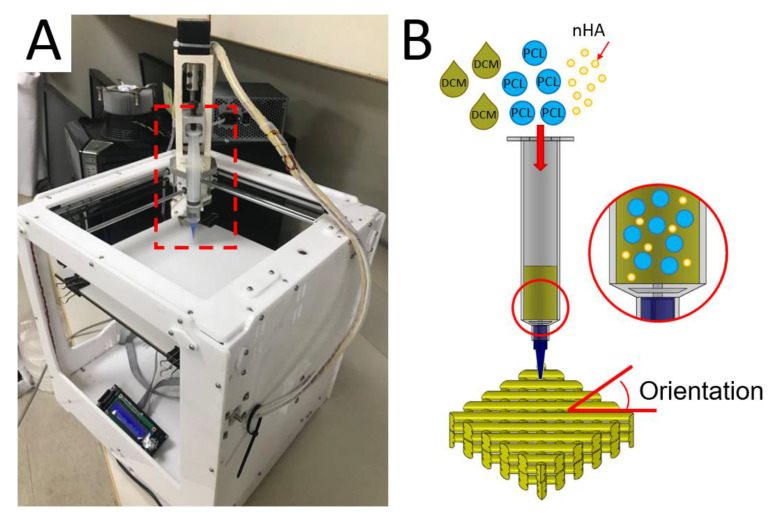
(**A**) Image of the solution-extrusion three-dimensional (3D) printer and (**B**) the solution printing of polycaprolactone/nano-hydroxylapatite (PCL/nHA) composites with desired orientation.

The 3D printing experiments were completed on a lab-made device (Figure 1A), which consists of an extrusion feeder, steering step motors, a syringe with a delivering nozzle (inner diameter: 180 μm), a collection table, and a control port connected to a computer. An open control Cura code was used to monitor the entire printing course.

To print nanocomposite parts, PCL (2.5 g) and nHA (0.133 g) were fixed with DCM based on distinct weight-to-volume ratios and stirred for 3 h. The mixed solution was then added to the extruding feeder for printing (Figure 1B). In the 3D printing process, the delivery nozzle was actuated by a computer-commanded step motor, thus extruding and layering the PCL/nHA solution on the collection table. As soon as the solvent evaporated, strips of PCL/nHA (about 0.2 mm in thickness) were laid on the table in successive layers.

### 2.3. Processing Variables

The effect of distinct processing variables on the tensile strengths of printed PCL/nHA specimens was investigated. Four variables were chosen: (1) PCL/nHA-to-DCM ratio, (2) fill density, (3) orientation of the extruded strips (Figure 1B), and (4) moving speed of the delivering nozzle. A few test trials were first completed to identify the ranges of processing values able to successfully print the nanocomposite specimens. The ratios of PCL/nHA to DCM were 2.5 g/0.133 g:5.8 mL, 2.5 g/0.133 g:6.0 mL, 2.5 g/0.133 g:6.2 mL, and 2.5 g/0.133 g:6.4 mL (*w*/*v*). Meanwhile, the fill densities were 50%, 55%, 60%, and 65%. The shifting speeds of the delivering nozzle spanned from 30 to 60 mm/s. The orientations of extruded strips were 45°, 60°, 75°, and 90°. The variables and variable values used for the experiment are listed in Table 1. After printing, the specimens were placed in an oven at room temperature for 72 h to completely vaporize the solvents.

**Figure 2:**
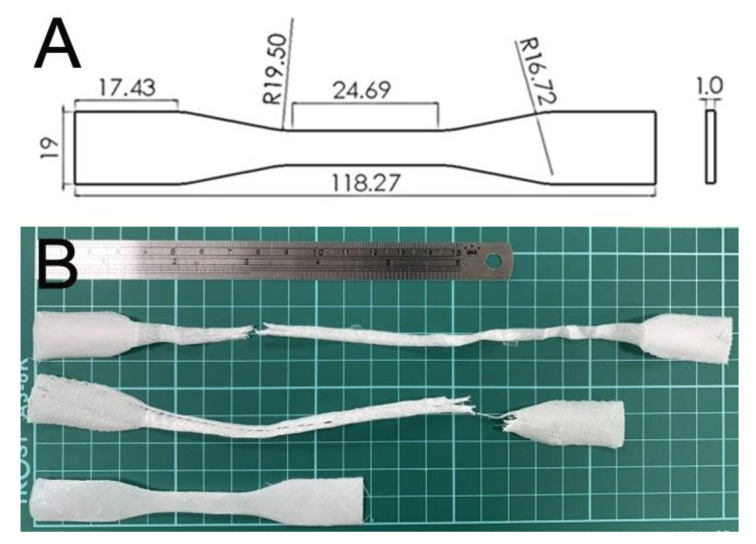
(**A**) Layout and dimensions of the samples and (**B**) ruptured specimens.

A dumbbell geometry (Figure 2A) was used to 3D print the test parts. The code used to control the migration of the dispensing nozzle was established using commercial software from Solidworks (Waltham, MA, USA). Post-printing, the tensile strengths of prepared PLC/nHA nanocomposites were assessed with a Lloyd test machine (Ameteck, Berwyn, PA, USA). The stretching rate for the specimens was 50 mm/min. The tensile strengths were calculated with the equation.

Strength (MPa) = Maximum load (N)/Part cross − sectional area (mm^2^) (1)

As shown in Table 1, one variable was varied every time while keeping the others constant (bold ones). The influence of every variable on the tensile strengths of the printed samples could be assessed. The experiment was repeated three times (N = 3) for every specimen.

### 2.4. Microscopic Examinations

The morphological structure of the 3D-printed samples was examined by a JEOL Model JSM-7500F FESEM (Tokyo, Japan) and an APEX-2010 profile projector (Taipei, Taiwan).

### 2.5. Printing of Drug-Eluting Screws

To demonstrate the capability of solution-extrusion 3D printing in fabricating drug- loaded implants, the optimum processing conditions obtained in previous sections were used to print the drug-eluting PCL/nHA screws. PCL (2500 mg), nHA (132 mg), and vancomycin and ceftazidime (312.5 mg each) were mixed with 6 mL of DCM. The solution was then used to print the screws.

### 2.6. Fourier-Transform Infrared Assay

The spectra of virgin PCL, nHA, PCL/nHA, and drug-loaded PCL/nHA were assessed employing a Nicolet iS5 Fourier-transform infrared (FTIR) spectrometer assay (Thermo Fisher, Waltham, MA, USA). The samples were first compressed as KBr discs for the assay, which was conducted at a resolution of 4 cm^−1^ and 32 scans. The spectra of the assay ranged from 400 to 4000 cm^−1^.

### 2.7. Differential Scanning Calorimeter Assay

The thermal properties of virgin PCL, PCL/nHA, and drug-loaded PCL/nHA were estimated by a TA-DSC25 differential scanning calorimeter (New Castle, DE, USA). The scanning temperature ranged from 30 to 350 °C while the specimens were heated at 10 °C/min.

### 2.8. In Vitro Release of PLC/nHA Screws

The elution patterns of vancomycin/ceftazidime from the drug-loaded PLC/nHA screws were assessed using an in vitro elution method [19]. Screws were put in glass tubes (one screw in each tube, N = 3) and filled with 1 mL of phosphate buffer solution (0.15 mol/L, pH 7.4). The tubes were kept in an isothermal oven at 37 °C for 24 h until the eluent was gathered and assayed. New phosphate buffer solution (1 mL) was added to the tubes for the next 24 h time interval. The process was duplicated for 14 days. The drug levels in the gathered eluents were characterized with Hitachi L-2200R multi-solvent high-performance liquid chromatography (HPLC) (Tokyo, Japan).

## 3. Results

### 3.1. Effects of Processing Parameters on Mechanical Strengths

PCL/nHA specimens were satisfactorily printed using solution-extrusion 3D printing. Figure 2B displays the fractured PCL/nHA nanocomposites post-tensile test. All specimens exhibited good ductile properties.

**Figure 3:**
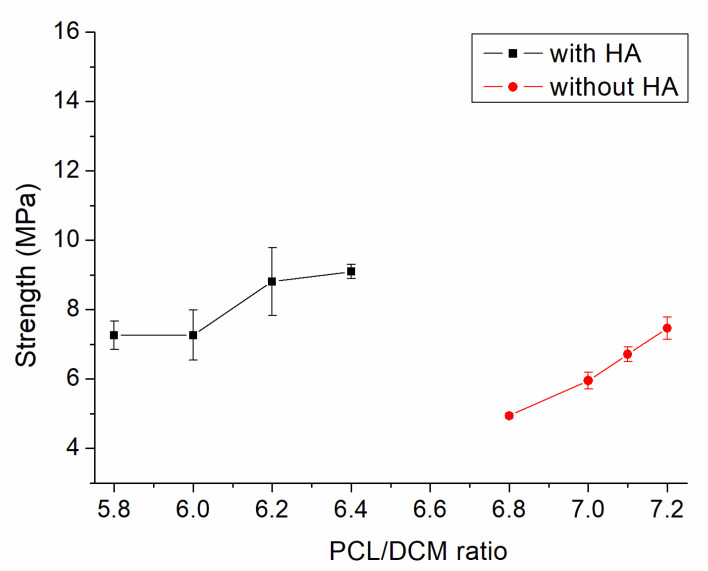
Influence of the polycaprolactone/dichloromethane (PCL/DCM) ratio on the tensile strengths of 3D-printed virgin PCL and PCL/nHA nanocomposite specimens.

**Figure 4:**
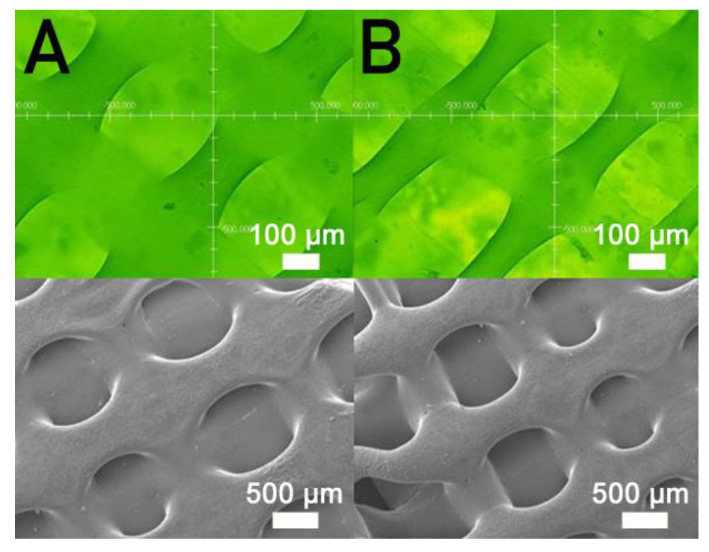
Profile projector (**top**) and scanning electron microscopy (SEM) (**bottom**) micro-photos of nanocomposite specimens printed with PCL/DCM ratios of (**A**) 2.5 g/6.4 mL and (**B**) 2.5 g/5.8 mL.

Figure 3 displays the tensile characteristics of 3D-printed PCL and PCL/nHA specimens. As expected, nHA-filled PCL parts showed superior tensile strengths to virgin PCL parts [18]. The results in Figure 3 also suggest that the maximum tensile strengths of PLC/nHA specimens improved as the concentration of DCM increased. Composite samples printed using a PCL/nHA:DCM ratio of 2.5 g/0.133g:6.4 mL showed the most superior mechanical strength, whereas printed specimens with a ratio of 2.5 g/0.133g:5.8 mL displayed the most inferior mechanical properties. Figure 4a,b shows the surface images of printed nanocomposite parts from the profile projector and SEM. The abundant solvent during the printing process helped promote healing at the extruded strip interfaces not only in the same layer, but also across layers. The tensile strengths increased accordingly.

**Figure 5:**
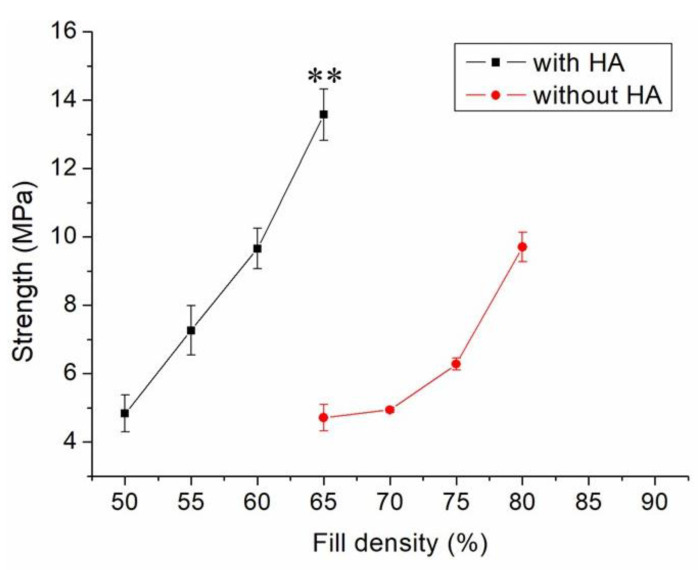
Effect of fill density on the tensile strengths of 3D-printed virgin PCL and PCL/nHA nanocomposite specimens (** *p* < 0.01, virgin PCL vs. PCL/nHA).

**Figure 6:**
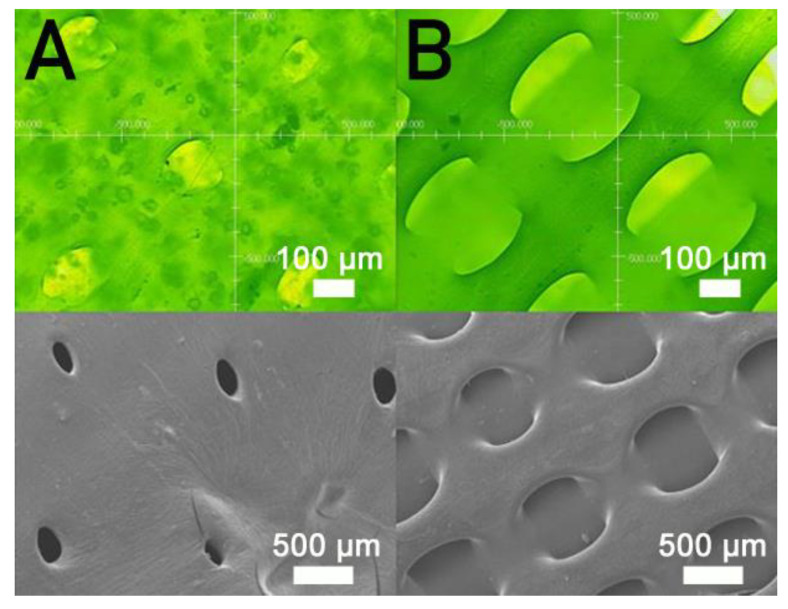
Profile projector (**top**) and SEM (**bottom**) micro-photos of nanocomposite specimens printed with fill densities of (**A**) 65% and (**B**) 50%.

Figure 5 shows the measured strengths of PCL/nHA specimens printed with different fill densities. The estimated ultimate tensile raised with the fill density. Figure 6 shows the micro-images of specimen surfaces printed with 65% and 50% fill densities. Imperfectly healed pores were observed on the surfaces of the printed specimens. Superior healing can be noted in Figure 6A (65% fill density) compared to Figure 6B (50% fill density). Since the specimens made with 65% fill density possessed the smallest pore sizes, they showed superior mechanical strengths. Meanwhile, nanocomposites printed with a lower fill density exhibited pores of greater sizes, resulting in stress concentrations in the tensile test process. Composite parts therefore possessed inferior mechanical properties.

**Figure 7:**
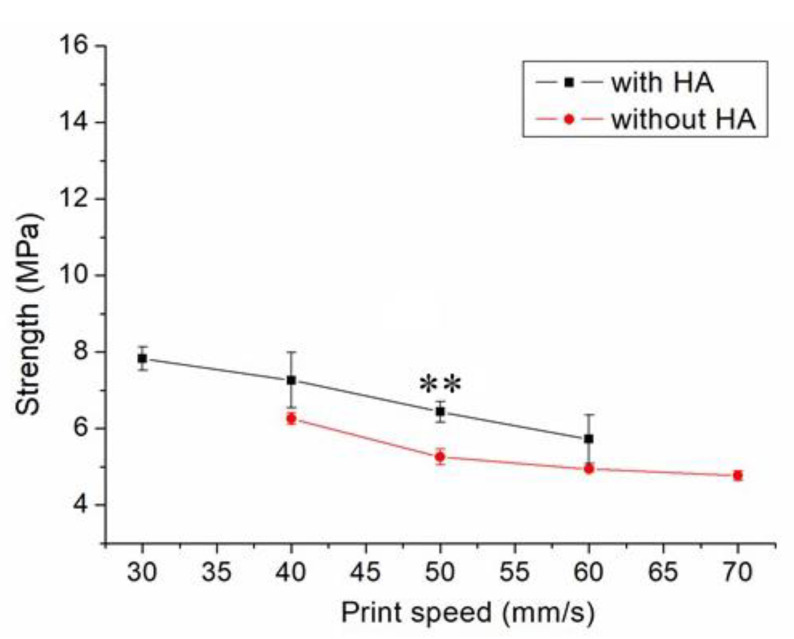
Influence of print speed on the tensile strengths of 3D-printed virgin PCL and PCL/nHA nanocomposite specimens (** *p* < 0.01, virgin PCL vs. PCL/nHA).

**Figure 8:**
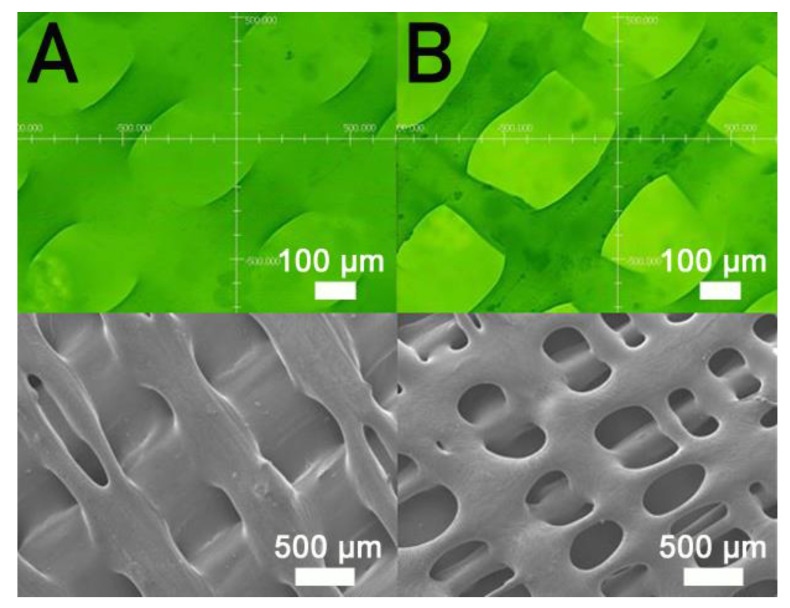
Profile projector (**top**) and SEM (**bottom**) micro-photos of nanocomposite specimens printed with nozzle speeds of (**A**) 30 mm/s and (**B**) 60 mm/s.

Figure 7 displays the influence of print speed on the ultimate strength of the printed nanocomposites. The ultimate strength decreased in general as the tip speed increased. Nanocomposite parts printed at a speed of 30 mm/s showed the highest strengths, and samples printed at a print speed of 60 mm/s displayed the most inferior strengths. Figure 8A,B shows the images of part surfaces printed using 30 and 60 mm/s, respectively. PCL/nHA nanocomposite parts printed at a speed of 60 mm/s possessed bigger pores than those printed with 30 mm/s. Small orifices were observed on the surface of 60 mm/s printed parts, which mainly resulted from incomplete healing. Manufactured 60 mm/s parts thus illustrated inferior mechanical strengths.

**Figure 9:**
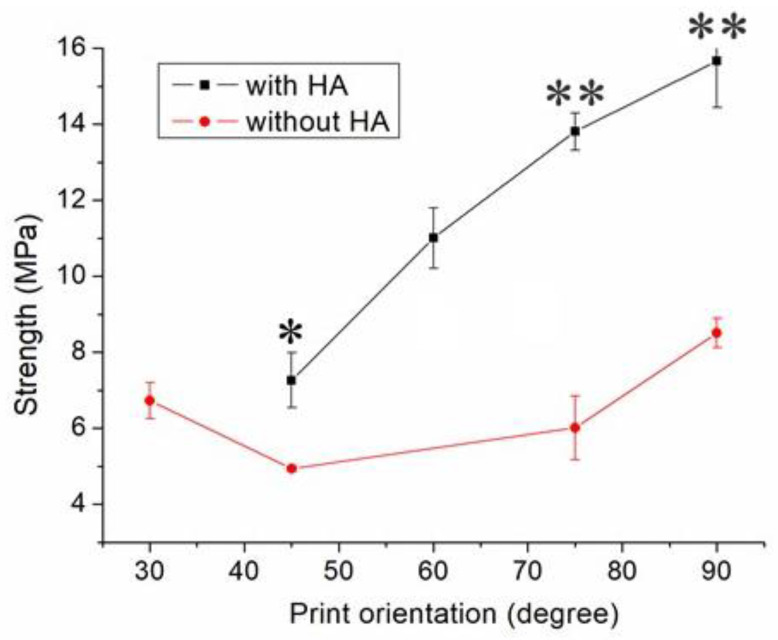
Influence of print orientation on the tensile strengths of 3D-printed virgin PCL and PCL/nHA nanocomposite specimens (* *p* < 0.05; ** *p* < 0.01, virgin PCL vs. PCL/nHA).

**Figure 10:**
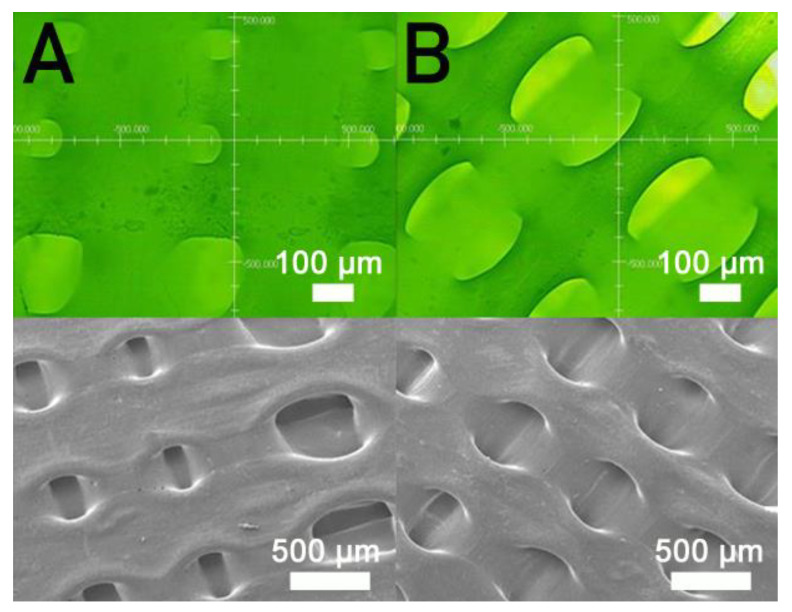
Profile projector (**top**) and SEM (**bottom**) micro-photos of nanocomposite specimens printed with orientations of (**A**) 90° and (**B**) 45°.

Figure 9 indicates the maximum tensile strength of these samples printed using different orientations of 45°, 60°, 75°, and 90°, suggesting that the nanocomposite specimen printed at a 90° orientation showed the most superior tensile strength, and those at 45° led to the least mechanical strengths. Figure 10A,B shows the images of part surfaces printed with orientations of 90° and 45°. Clearly, 45° printing led to less healing and larger pores on the parts’ surfaces. As these specimens are exposed to foreign loads, stress can happen and result in ruptures. Printed part strengths thus diminish under these loads.

### 3.2. Drug Release from Printed Implants

Drug-eluting screws were prepared using the optimum processing conditions obtained in Section 3.1, namely a PCL/DCM ratio of 2.5 g/0.133 g:6.4 mL, a fill density of 65%, a nozzle shifting speed of 30 mm/s, and a 90° printing orientation. The ultimate strength and elastic modulus thus obtained were 15.67 ± 1.22 and 37.49 ± 1.36 MPa, respectively.

**Figure 11:**
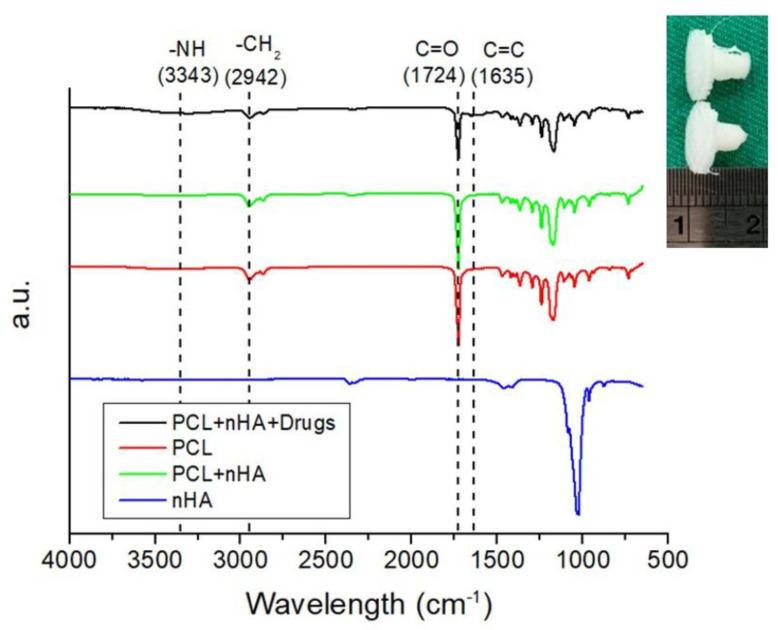
Fourier-transform infrared (FTIR) spectra of virgin PCL, nHA, PCL/nHA, and drug-loaded PCL/nHA nanocomposites (**upper right** is a photo of 3D-printed drug loaded PLC/nHA screws).

Figure 11 displays the Fourier-transform infrared (FTIR) spectra of pure PCL, PCL/nHA, and drug-loaded PCL/nHA screws. The vibration peak of PO_4_^3−^ near 1040 cm^−1^ for nHA diminished after the material was incorporated into the PCL matrix [20]. The peaks at 1724 and 1635 cm^−1^ may be attributed to the C=O and C=C bonds, respectively, of the incorporated drugs. The vibration peak at 2942 cm^−1^, corresponding to a CH_2_ bond, was promoted due to the addition of vancomycin. Additionally, the vibration at 3340 cm^−1^ resulted from the N–H bond of the anti-microbial agents [21,22].

**Figure 12:**
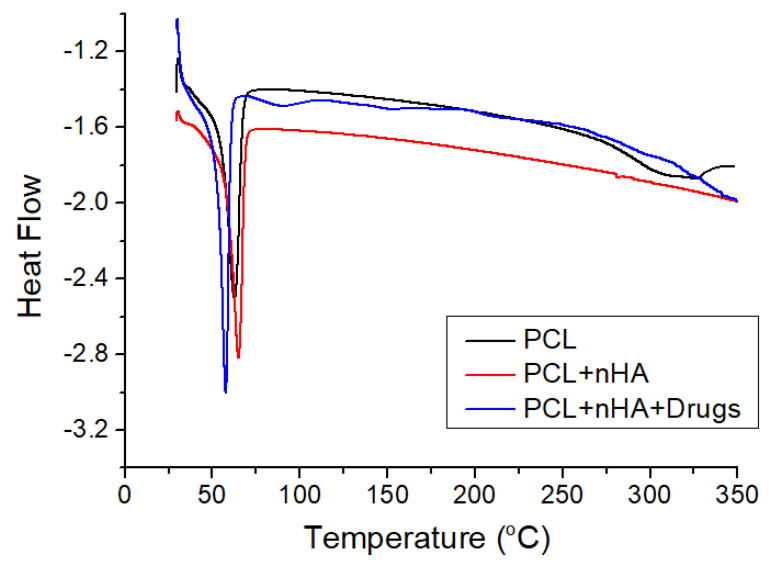
Differential calorimetry scanning (DSC) curves of virgin PCL, PCL/nHA, and drug-loaded PCL/nHA composites.

The thermal properties of virgin PCL, PCL/nHA, and drug-loaded PCL/nHA screws were assessed, and the results are displayed in Figure 12. Although the incorporation of nHA caused a slight increase in the melting point of pure PCL from 62.78 to 65.02 °C, the addition of drugs tended to reduce the melting temperature of 3D-printed PCL/nHA screws to 57.75 °C.

**Figure 13:**
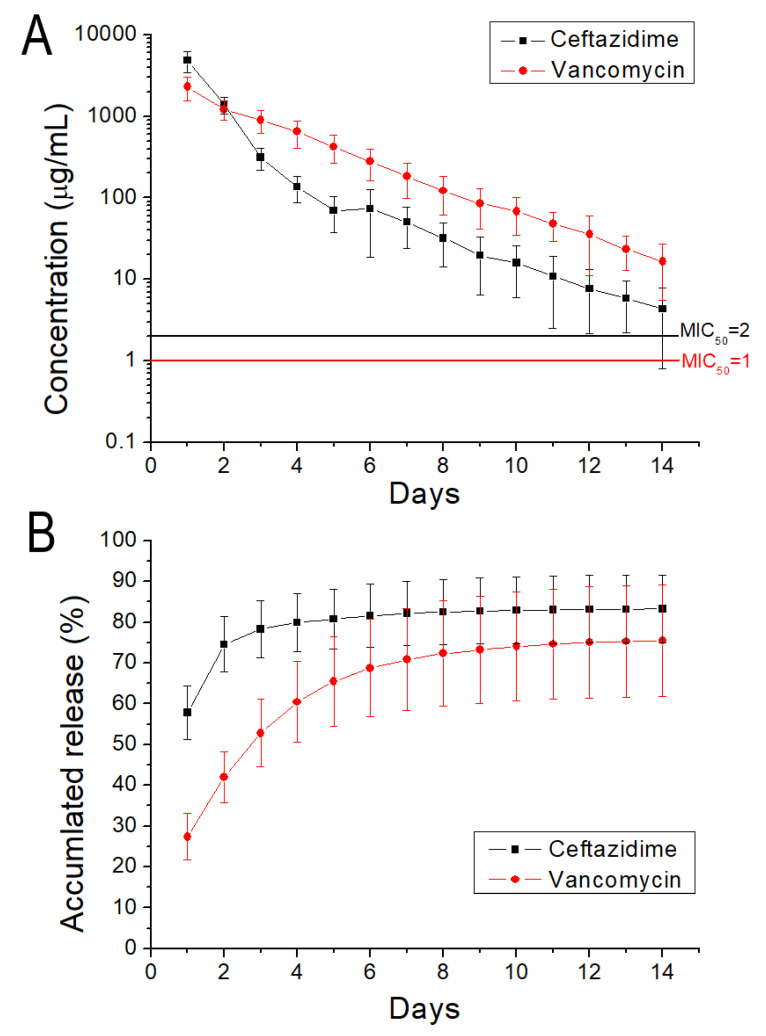
(**A**) Daily and (**B**) cumulative release of vancomycin and ceftazidime from PCL/nHA screws.

The release of antibiotics from the 3D-printed screws was characterized. Figure 13 displays the daily and cumulative releases of vancomycin and ceftazidime. A burst release was noticed for the anti-microbial agents at day 1, after which a steady and diminishing elution of pharmaceuticals was observed. The drug-loaded PCL/nHA screws could elute high levels of vancomycin and ceftazidime (higher than the minimum inhibitory concentrations) for more than 14 days. Antibiotic levels were maintained at a high level after the 3D solution-extrusion printing procedure, demonstrating that the 3D printing did not inactivate the antimicrobial agents during the fabrication process.

## 4. Discussion

This study explored the effect of distinct printing variables on mechanical strengths of printed PCL/nHA materials. PCL pertains to a biodegradable polymer material that resorbs gradually through hydrolysis [23], whereas HA is a primary component for hard tissues, including bone and teeth, and has been widely used for bone repair, bone augmentation, and implant coatings [24]. The mechanical properties of a material represent its response to an externally applied load, and are one of the most important basic characteristics of a good design. Two factors might have influenced the mechanical strengths of our 3D-printed nanocomposites and helped decide the success of the composites for a specific application. The primary factor pertained to the healing/sealing of extruded materials, and the next factor was the morphological structure of printed products.

The first factor that affected the final product property was the healing and chain entanglement at the inter-boundaries of polymeric strips. In 3D printing, solvent vaporization and molecular chain diffusion occur at the solidification stage of polymer materials. Promotion of molecular entanglement at the interface of extruded strips is required and accounts for the eventual tensile characteristics of 3D-printed samples [25,26]. The optimum status tends to be a semi-dilute and moderately entangled mode that arises at the critical entanglement concentration [27]. The period is a crossover point between a semi-diluted unentangled mode and a semi-diluted moderately entangled mode. Promoted entanglement and the associated strips sealing at the interfaces are thus expected. With regard to the second factor, a solution-extrusion 3D-printed part may possess irregular morphology owing to imperfectly healed pores or flaws that result in stress concentrations as exposed to foreign loads. This process may in turn cause deterioration of the tensile strengths of printed nanocomposites.

The empirical outcomes in Figure 3 indicate that the measured tensile strengths of 3D-printed nanocomposites increased with the volume fraction of the solvent. The nanocomposites prepared with the PCL/nHA-to-DCM ratio of 2.5 g/0.133g:6.4 mL exhibited the greatest tensile strengths. When a small amount of DCM was used, the solvent may have evaporated too fast, resulting in lack of time for chain entanglement at the interfaces of extruded strips. An abundant concentration of DCM kept the polymers within a semi-diluted status for a longer period of time and promoted strip healing/sealing at the inter-boundaries, either within the current layer or across distinct layers (Figure 4). Printed materials therefore illustrate superior strengths.

Figure 5 shows that the estimated tensile strengths of nanocomposites increased as the fill density was increased. Fill density represents the quantity of polymer nanocomposites used in manufacturing a specimen. A greater fill density represents potent polymeric materials inside printed specimens, thereby leading to a more superior part. Additionally, fulfilling the specimens with extra materials also resulted in pores of smaller sizes (Figure 6). Printed product quality increased accordingly.

Figure 7 implies that mechanical strength declined when print speed decreased. Following the solution extrusion from the delivering nozzle, the solvent started to vaporize. When the print speed was excessively high, not enough time was allowed for molecular entanglement of the polymers across the strip interfaces (Figure 8). Additionally, the solvent may not have had sufficient time to vaporize and may then have diffused and disintegrated the surrounding strips. Chain entanglement and the relevant part strengths diminished accordingly.

Nanocomposite specimens printed with a 45° orientation demonstrated the most inferior part strength, and 90° printed parts showed the highest strengths (Figure 9). This finding might be due to the fact that the 90° oriented strips restricted the quantity of solvent that vaporized quickly. Abundant time was allowed for the polymer solution to achieve chain entanglement across strip boundaries (Figure 10A). The tensile properties of 3D-printed samples thereby increased. Additionally, the printed 45° orientated nanocomposite parts presented a greater number of pores (Figure 10B). When stretched by the external tensile forces, these large-size pores possessed greater chances of undergoing damage from stress. Printed parts therefore displayed the most inferior tensile properties.

Finally, this study successfully developed antimicrobial agent-loaded PCL/nHA screws using the solution of extrusion 3D printing technology. After 3D printing, some drugs may have remained at the surface of the screws, thus leading to a burst release when the screws were submerged in a PBS solution. After the burst release, the release mechanism was mainly controlled by channel diffusion. When the loading of antibiotics was low, the pharmaceuticals would have been separated in the polymer matrix. The drugs may not have been capable of penetrating the matrix at an effective rate. When the pharmaceutical loading was further increased, the drugs may have bonded together to create channels transmitting to the surface of the 3D-printed parts [18]. A steady and slow release of the drugs was thus observed after the burst release. The experimental results demonstrate that the drug-loaded PCL/nHA screws can offer extended elution of high concentrations of vancomycin/ceftazidime (superior to the minimum inhibitory concentrations) for more than 14 days. All of these findings demonstrate the great potential of solution-extrusion 3D printing for the manufacture of drug-loaded implants.

## 5. Conclusions

This study explored the solution-extrusion 3D printing of vancomycin- and ceftazidime-loaded PCL/nHA materials utilizing a lab-made printer. The influence of distinct parameters on the printed part quality was examined. The empirical outcomes illustrate that the tensile property of printed nanocomposites increases with the fill density yet diminishes with a decrease in the ratio of PCL/nHA to DCM and print speed. Nanocomposite parts printed with a 90° orientation demonstrated the most superior mechanical properties. In addition, the drug-loaded PCL/nHA screws can provide extended elution of high levels of vancomycin/ceftazidime over a 14-day period. Eventually solution-extrusion 3D printing technology may be used to print drug-loaded implants for various medical applications.

## Figures and Tables

**Table 1:** Variables utilized for the three-dimensional (3D) printing of PCL/nHA parts.

Variable	A: PCL/nHA to DCM Ratio (*w*/*v*)	B: Fill Density (%)	C: Print Speed (mm/s)	D: Print Orientation
Level 1	2.5 g/0.133g:5.8 mL	50	30	45°
Level 2	**2.5 g/0.133g:6.0 mL**	**55**	**40**	**60°**
Level 3	2.5 g/0.133g:6.2 mL	60	50	75°
Level 4	2.5 g/0.133g:6.4 mL	65	60	90°
